# What Happens When the Patient Doesn’t Die? Understanding Live Discharge from Hospice Care

**DOI:** 10.1093/geroni/igab046.3550

**Published:** 2021-12-17

**Authors:** Stephanie Wladkowski, Kathryn Coccia, Anna Wingo, Ruaa Al-Juboori, Cara Wallace

**Affiliations:** 1 Eastern Michigan University School of Social Work, Eastern Michigan University, Michigan, United States; 2 Saint Louis University, St. Louis, Missouri, United States; 3 Saint Louis University, Saint Louis University, Missouri, United States; 4 Saint Louis University, Saint Louis, Missouri, United States

## Abstract

Hospice has been shown to improve end-of-life outcomes, yet with eligibility limited to a six-month prognosis, the hospice system is not structured to meet longer-term needs. Though hospice is strongly associated with death, some enrolled patients do not decline as predicted leading to what is referred to as a ‘live discharge.’ In 2018, 6.3% of all hospice discharges were patients discharged alive due to decertification, or no longer meeting eligibility requirements. The aim of this presentation is to review current literature surrounding live discharge, discuss policy and practice challenges within current discharge practices, and present new research directions from two current NIH-funded studies. Studies of live discharge often do not differentiate between revocation and decertification, yet these are very different phenomena, particularly regarding decision making. Patients discharged from hospice are often referred to as “not dying fast enough,” or “failure to die on time,” yet, they are still dying from chronic illness, just outside the prescribed six-month framework. Affected patients lose access to important supportive services and resources, still require substantial care, and can struggle to process feelings of abandonment and uncertainty. Further, an increased burden is placed on primary caregivers who may be unprepared for this transition. Clinicians across agencies report great variability in managing live discharges with no standardized protocols. These findings demonstrate the complexities of live discharge, the need for more research to support a standardized and reimbursable discharge process and to define unmet needs for both patients and caregivers affected by live discharge.

